# Gene Variants in Components of the microRNA Processing Pathway in Chronic Myeloid Leukemia

**DOI:** 10.3390/genes15081054

**Published:** 2024-08-10

**Authors:** Guillermina Chavaro-Francisco, Araceli Hernández-Zavala, Camila E. Bravo-Cidro, Sandybel Rios-Rodriguez, Mabel Muciño-Sánchez, Marisol López-López, Xóchitl H. Castro-Martínez, Irma Olarte-Carrillo, Anel Garcia-Laguna, Gilberto Barranco-Lampón, Adrián De la Cruz-Rosas, Adolfo Martínez-Tovar, Emilio J. Córdova

**Affiliations:** 1 Section of Research and Postgraduate Studies, Superior School of Medicine, National Institute Polytechique, Mexico City 11340, Mexico; guille.chavaro94@gmail.com (G.C.-F.); araheza17@gmail.com (A.H.-Z.); 2 Oncogenomics Consortium Laboratory, Clinic Research Department, National Institute of Genomic Medicine, Mexico City 14610, Mexico; 2173063326@alumnos.xoc.uam.mx (C.E.B.-C.); faty.rios17@gmail.com (S.R.-R.); mamusa569@gmail.com (M.M.-S.); 3 Department of Biological Systems, Metropolitan Autonomous University, Campus Xochimilco, Mexico City 04960, Mexico; marisollopezlopez@gmail.com; 4 School of Biology, Metropolitan Autonomous University, Campus Xochimilco, Mexico City 04960, Mexico; 5 Genomics of Psychiatric and Neurogenerative Diseases Laboratory, National Institute of Genomic Medicine, Mexico City 14610, Mexico; xcastro@inmegen.gob.mx; 6 Molecular Biology Laboratory, Service of Hematology, Hospital General de Mexico, “Dr. Eduardo Liceaga”, Mexico City 06720, Mexico; irmaolartec@yahoo.com (I.O.-C.); gala.anel@gmail.com (A.G.-L.); drgibalampon@gmail.com (G.B.-L.); mtadolfo73@hotmail.com (A.M.-T.)

**Keywords:** chronic myeloid leukemia, microRNAs biogenesis, gene variants, Hasford score

## Abstract

Current therapy in chronic myeloid leukemia (CML) has improved patient life expectancy close to that of healthy individuals. However, molecular alterations other than BCR::ABL1 fusion gene in CML are barely known. MicroRNAs are important regulators of gene expression, and variants in some of the components of microRNA biosynthesis pathways have been associated with genetic susceptibility to different types of cancer. Thus, the aim of this study was to evaluate the association of variants located in genes involved in the biogenesis of microRNAs with susceptibility to CML. Fifteen variants in eight genes involved in the biogenesis of miRNAs were genotyped in 296 individuals with CML and 485 healthy participants using TaqMan probes. The association of gene variants with CML and clinical variables was evaluated by a Chi-square test, and odds ratios and 95% confidence intervals were estimated by logistic regression. The variant rs13078 in *DICER1* was significantly higher among CML individuals than in healthy participants. In addition, the variants rs7813 and rs2740349 were significantly associated with worse prognosis, according to their Hasford scores, whereas the rs2740349 variant was also associated with a later age at diagnosis. These findings suggest that variants in components of the microRNA biogenesis pathway could be involved in CML genetic risk.

## 1. Introduction

Chronic myeloid leukemia (CML) is a myeloproliferative malignant disease characterized by the presence of an aberrantly short chromosome 22, named the Philadelphia chromosome. This altered chromosome is the product of reciprocal translocation t(9;22)(q34;q31) and results in the fusion of the *BCR* gene in chromosome 22 with the *ABL1* gene located in chromosome 9 [1]. The *BCR::ABL1* fusion gene encodes a chimeric protein with the exacerbated activity of tyrosine kinase, which is capable of activating different intracellular signaling pathways, including RAS-MAPK, JAK-STAT, and SRC [2].

The characterization of the *BCR::ABL1* fusion gene as the main molecular alteration underlying CML development allowed the design of the first successful target therapy for malignant diseases. This therapy was based on the use of Imatinib, a highly specific tyrosine kinase inhibitor (TKI), which was significantly more efficient than Interferon, the conventional therapy used in the past in the treatment of CML [3]. At present, five other TKIs have been approved by the FDA for CML therapy, including nilotinib, dasatinib, bosutinib (second generation), ponatinib (third generation), and asciminib (fourth generation) [4]. Currently, the use of TKIs as the first-line therapy in CML has significantly increased 10-year survival rates from 20% to 85% [4]. Although a small group of CML patients (10–15%) show resistance or intolerance to TKIs, most CML patients exhibit life expectancies similar to those found in healthy individuals [5].

In contrast to the great advancement in the treatment of CML, worldwide incidence of this disease has remained unchanged, and as a consequence of the success of TKI therapy, its prevalence has significantly increased in the last few years. In this sense, it was estimated that in 2017, about 15 million people were living with CML around the world [6]. Risk factors associated with CML, including genetic factors, are not widely known, with high exposure to radiation and benzene as the only recognized factors related to CML development [7]. The identification of the genetic factors associated with increased CML risk could help in the establishment of public health strategies that allow the prevention of this disease with a consequent reduction in its prevalence.

MicroRNAs (miRNAs) are short, non-coding RNAs of 19–22 nucleotides long that post-transcriptionally regulate gene expression by directly binding to protein-coding transcripts [8]. MiRNAs interact with their target transcripts through base pair complementarity binding between a short sequence located at the miRNA 5′ end, named the seed region, and a canonical sequence situated in the 3′ untranslated region (3′ UTR) of the target transcript. Since the seed regions in miRNAs are very short sequences, each individual miRNA can regulate different transcripts, and in the same sense, an individual transcript can be regulated by different miRNAs [9,10]. At present, more than 2600 mature sequences of miRNAs have been deposited in an international database reservoir called miRBase [10], and it has been estimated that about 30% of coding genes might be regulated by miRNAs [11].

MiRNAs regulate several cellular processes, including proliferation, survival, and differentiation [12]. Accordingly, alterations in the expression of miRNAs are a common event in different types of cancers. For instance, the up-regulation of miR-130b-5p and miR-222-3p has been reported in breast cancer [13], whereas the down-regulation of miR-148a and miR-152 has been observed in gastric [14,15] and bladder cancers [16]. In addition, genetic studies have revealed that the aberrant expression of miR-126 plays a critical role in the leukemogenesis and progression of acute myeloid leukemia [17]. Particularly in CML, miR-125a-3p and miR-320b are differentially expressed in CML patients [18]. In an independent study, the down-regulation of miR-342-5p expression was found to be associated with CML progression [19]. Taken together, these findings indicate the importance of miRNA regulation in different types of cancers, including leukemia.

The biogenesis of miRNAs is a highly regulated multi-step process, starting with its transcription by RNA polymerase II as long hairpin structures called primary miRNAs (pri-miRNAs). Since miRNAs can be transcribed as monocystronic (one gene) or polycistronic (multiple genes) transcripts, the sizes of pri-miRNAs are highly diverse. Later, pri-miRNAs are cleaved into precursor miRNAs (pre-miRNAs) of ~65–70 nucleotides long by a type III ribonuclease named DROSHA in complexes with DGCR8 proteins. Then, pre-miRNAs are transported into the cytoplasm by the Exportin-5 (XP05)–RAN complex and further processed into ~22 nucleotide mature duplex RNA molecules, by another type III ribonuclease called DICER1 in complexes with the TRBP protein. This mature duplex miRNA are composed of a passenger and a guide strand. The passenger miRNA strand is usually degraded, whereas the guide miRNA strand is loaded into the RNA-induced silencing complex (RISC) by the proteins AGO1-4 and GEMIN3-4 [20].

Recent studies have demonstrated the importance of gene variants in components of the biosynthesis process of miRNAs as risk factors for different human diseases. For instance, variants rs7813 in *GEMIN4* and rs636832 in *AGO1* have been associated with an increased risk of the multiple sclerosis variant [21], whereas variant rs13078 in *DICER1* has been associated with a decreased risk of type 2 diabetes mellitus [22]. In addition, the variants rs197388 in *GEMIN3* and rs3744741 in *GEMIN4* have been associated with an increased risk of depression [23]. In the case of malignant diseases, the variants rs7157322 and rs3742330 in the *DICER1* gene have been associated with gastric adenocarcinoma and papillary thyroid cancer, respectively [24,25]. Similarly, variants rs3742330 in *DICER1*, rs11077 in *XPO5*, and rs10719 in *DROSHA* are associated with colorectal cancer risk [26]. Importantly, functional assays have demonstrated that variant rs10719, located in the 3’ untranslated region of *DROSHA*, decreases the binding of hsa-miR-27b with the consequent elevation of *DROSHA* transcripts [27].

Currently, no studies about the participation of variants in miRNA biogenesis and CML development have been performed. Since alterations in the expression of miRNAs are found in CML, variants in genes involved in the biogenesis of miRNAs could be associated with this disease. Thus, the aim of the present study was to analyze the association of gene variants in components of the miRNA machinery pathway and the development of CML.

## 2. Material and Methods

### 2.1. Study Population

Our sample population was composed of a total of 781 unrelated subjects, comprising 296 individuals previously diagnosed with CML and 485 unrelated healthy persons. Case and control subjects were recruited from tertiary hospitals in Mexico City. The case group was composed of 170 (57%) males and 126 (43%) females, whereas the control group comprised 271 (56%) males and 214 (44%) females. The median ages in the case and control groups were 45 ± 14.7 and 48 ± 7.4, respectively. CML was initially diagnosed by a hematological test and then confirmed through the detection of translocation t(9;22)(q34;q11) by conventional cytogenetics, or the detection of *BCR::ABL1* transcripts by qPCR. All participants with CML were of recent diagnoses and all of them were in the chronic phase of the disease.

This study was conducted in accordance with the Declaration of Helsinki and approved by the Research and Ethics Committees of the National Institute of Genomic Medicine (Registry number 13/2019/1). All participants signed a written informed consent form.

### 2.2. Gene Variant Genotyping

Genomic DNA was extracted from 10 mL of circulating blood samples using the Puregene blood kit following manufacturer instructions (Qiagen, Hilden, Germany). Sample genotyping was performed using commercial TaqMan probes as follows: rs2293939 (C_1707002_10), rs4961280 (C_11609859_10), rs720012 (C_2255497_30), rs3742330 (C_27475447_10), rs13078 (C_7504801_10), rs10719 (C_7761648_10), rs6877842 (C_1153852_10), rs197388 (C_32363724_30), rs197414 (C_923336_10), rs7813 (C_2988802_40), rs2740349 (C_2988801_1), rs4968104 (C_22274226_10), rs9611280 (C_29619916_10), rs11077 (C_3109165_1), and rs34324334 (C_25933077_10) (Applied Biosystems, Foster City, CA, USA). PCR amplification for allelic discrimination was carried out in a QuantStudio 3 real-time PCR and analyzed in the Design & Analysis Software V. 2.8.0 (Applied Biosystem, Foster City, CA, USA). The genotyping call rate exceeded 95%. Approximately 20% of the samples were randomly genotyped twice, with concordance above 98%.

### 2.3. Statistical Analysis

Deviation from the Hardy–Weinberg equilibrium was determined by a Chi-square test. To evaluate the association of variants in components of the miRNA biogenesis process with CML and clinical variables, Chi-square and Fisher’s tests were performed. Additionally, odds ratios (ORs) and 95% confidence intervals (CIs) were estimated using a logistic regression model. Linear regression was used to evaluate the effect of gene variants on age at CML diagnosis (β value). Both methods were adjusted by age and sex. All statistical analyses were performed with the PLINK software 1.9 [28]. A *p*-value less than 0.05 was considered as the threshold of statistical significance.

## 3. Results

### 3.1. Characteristics of the Study Population

The demographic and clinical characteristics of CML patients and healthy individuals are shown in Table 1. From the 296 individuals diagnosed with CML, clinical data for Sokal and Hasford scores were obtained from 118 participants, whereas clinical data for EUTOS scores could only be obtained from 97 participants. Based on Sokal scores, 44% of patients were classified as high risk, 29% as intermediate risk, and 27% as low risk. Regarding Hasford scores, 41% of patients were classified as high risk, 30% as intermediate risk, and 29% as low risk, respectively. According to EUTOS scores, 52% were classified as high risk and 48% as low risk (Table 1).

### 3.2. Gene Variants in Components of miRNA Biosynthesis Pathway and CML

After genotyping the 15 studied variants in the case and control groups, the minor allele frequency of rs197414 in *GEMIN3* and rs4968104 in *GEMIN4* significantly deviated from the Hardy–Weinberg equilibrium (*p* < 0.05). Thus, both variants were excluded from further analysis. The rest of the variants showed no deviation from the Hardy–Weinberg equilibrium. In the association study, the frequency of the minor allele of rs13078 in *DICER1* was significantly higher in individuals affected with CML in comparison to healthy subjects (0.09 vs. 0.06; *OR* = 1.64, 95% CI [1.105–2.421], *p* = 0.013; Table 2). Moreover, the number of participants carrying the minor homozygote genotype of this variant was significantly higher among the cases compared to the controls (0.034 vs. 0.002; OR = 15.06, 95% CI [2.824–373.6]; Table 3). It is worth noting that no significant differences were observed in the number of individuals carrying the heterozygote genotype of this variant among the cases and controls, suggesting a recessive effect. The allele and genotype frequencies of the rest of the studied variants showed no significant differences between the cases and controls (Table 2 and Table 3).

### 3.3. Association of Variants in Components of miRNA Biogenesis with Prognostic Scores and Age at Diagnosis in CML

To analyze the participation of SNVs in miRNA biogenesis pathways with CML progression, the association of the studied variants with the prognostic scores of EUTOS, Sokal, and Hasford was evaluated. For comparison purposes, participants with low and middle risk in the Sokal and Hasford scores were grouped together with participants classified as low risk in the EUTOS scores. For the comparison of participants with high risk in the three prognostic scores against individuals with low risk for EUTOS and low and middle risk in the Sokal and Hasford scores, the frequency of minor alleles of the variants rs7813 and rs2740349 in *GEMIN4* was higher in the high-risk group than in the low- and middle-risk group (Sokal: 0.36 vs. 0.26 and 0.25 vs. 017; Hasford: 0.39 vs. 0.24 and 0.27 vs. 0.14; and EUTOS: 0.35 vs. 0.26 0.23 vs. 0.17, respectively). However, only in the case of the Hasford score was this difference statistically significant for both variants (rs7813: OR = 1.96, *p* = 0.019 and rs2740349: OR = 2.23, *p* = 0.015, respectively). None of the other studied variants showed any association with CML prognostic scores (Table 4).

The impact of variants in miRNA biosynthesis on age at diagnosis was also evaluated. In this analysis, CML individuals carrying the variant allele of rs2740349 in *GEMIN4* showed a significantly later age at diagnosis than CML participants with the wild-type allele (~5 years, *p* = 0.003; Table 5). The variant rs7813, also present in *GEMIN4*, showed a tendency to associate with a later age at diagnosis (~3 years, *p* = 0.059; Table 5). None of the other variants showed any association with age at diagnosis.

## 4. Discussion

MiRNAs are important regulators in different cellular processes, including proliferation, apoptosis, and cellular differentiation. Accordingly, alterations in the expression of miRNAs are frequently found in a variety of human illnesses, including cancer, diabetes, and cardiovascular diseases [12,13,14,15,16]. Importantly, recent studies have demonstrated that genetic variants in components of miRNA biogenesis pathways could be involved in the development of several types of cancer and metabolic diseases [21,22,23,24,25].

In this study, the association of 15 SNVs in eight genes involved in the biosynthesis process of miRNAs with CML susceptibility was evaluated. As important observations, the frequency of the minor allele and the minor homozygote genotype of the variant rs13078 in *DICER1* was significantly higher among CML individuals than in healthy participants. Additionally, the frequency of the minor alleles of *GEMIN4* variants rs7813 and rs2740349 was higher in the group of CML patients with high risk in comparison to the intermediate- and low-risk group according to their Hasford, EUTOS, and Sokal scores; however, this difference was significant only in the case of the Hasford score. Considering that all CML participants included in this study were in the chronic phase of the disease, this finding indicates that variants rs7813 and rs2740349 could be associated with a worse prognosis. Finally, the presence of the variant allele of rs2740349 was significantly associated with a later age at diagnosis of CML in comparison to noncarriers, whereas the variant rs7813 showed only a small association.

The *DICER1* gene encodes a protein of approximately 218 kDa with the activity of type III ribonuclease involved in the regulation of several small non-coding RNA pathways [29]. Alterations in the expression of *DICER1* have been found in different types of cancer such as breast carcinoma, hepatocellular carcinoma, and lung cancer [30,31,32]. Moreover, germline mutations in the *DICER1* coding region, which affects protein activity, have been related to a rare autosomal dominant genetic disorder named DICER syndrome [33,34]. This syndrome influences different benign and malignant tumors characterized by *DICER1* loss of function [35,36]. Moreover, in papillary thyroid cancer samples, pathogenic somatic variants in the *DICER1* gene are associated with a reduction in the abundance of 5p-derived miRNAs [37]. In this sense, common variants in *DICER1*, such as rs13078, rs105703, and rs3742330, have been associated with a variety of human illnesses, including cancer, diabetes, cardiovascular, autoimmune, and neurodegenerative diseases [22,38,39,40,41].

The rs13078 variant is located at the 3′UTR region of the gene, and it has been previously associated with decreased risk of type-2 diabetes [22] and increased risk of gestational hypertension, idiopathic male infertility, hepatocellular carcinoma, and larynx cancer [42,43,44,45]. A previous report demonstrated the direct binding of miR-892a to the 3′UTR of *DICER1* transcripts in a human laryngeal-cancer-derived cell line with a consequent reduction in DICER1 protein levels [46]. In this sense, variant rs13078 could modify the interaction of different miRNAs or RNA-binding proteins with *DICER1* transcripts, affecting the abundance of the protein and potentially affecting the global expression of miRNAs.

*GEMIN4* is a protein of approximately 120 kD, which is part of a multiprotein complex involved not only in miRNA biogenesis but also in the assembly and organization of the small nuclear ribonucleoproteins needed for mRNA splicing [47]. Recent studies have reported the association of germinal pathogenic variants in *GEMIN4* with an inherited neurodevelopmental disorder characterized by microcephaly, cataracts, and renal abnormalities [48,49]. An independent study also demonstrated the association of germinal variants with pediatric cataract syndrome [50]. Moreover, common variants in *GEMIN4*, such as rs7813, rs3744741, rs910924, and rs595961, have been associated with a variety of human diseases, including multiple sclerosis, depression, and cancer in different organs such as the stomach, lungs, and kidneys, among others [21,23,51,52,53].

The variants rs7813 and rs2740349 are missense variants (Arg1033Cys and Asp929Tyr, respectively). Although the variant rs2740349 has not been associated with any illnesses, previous studies have reported the association of rs7813 with several diseases, including multiple sclerosis, tuberculosis, gastric cancer, and lung cancer, among others [21,51,52,53,54,55]. Similar to our findings, the rs7813 variant was reported to be associated with the prognosis of esophageal squamous cell carcinoma [56].

This is the first study showing the association of variants in components of miRNA biogenesis pathways with CML and the progression of the disease. An important limitation of this study is the small number of CML individuals with available data for prognostic scores. However, our findings are in line with previous studies indicating the relevance of gene variants in miRNA biogenesis pathways in different types of malignant diseases. Further studies evaluating the impact of variants in miRNA biogenesis pathways on the abundance of miRNAs associated with CML are warranted.

## Figures and Tables

**Table 1:** Demographic and clinical characteristics of CML patients and healthy individuals.

	CML *n* = 296 (%)	Controls *n* = 485 (%)
Age, median ± SD	45 ± 14.7	48 ± 7.4
Males	170 (57%)	271 (56%)
Females	126 (43%)	214 (44%)
*Sokal Score (n = 118)*		
HR	52 (44%)	-
IR	34 (29%)	-
LR	32 (27%)	-
*Hasford Score (n = 118)*		
HR	48 (41%)	-
IR	36 (30%)	-
LR	34 (29%)	-
*EUTOS Score (n = 97)*		
HR	50 (52%)	-
LR	47 (48%)	-

SD: standard deviation, CML: chronic myeloid leukemia, HR: high risk, IR: intermediate risk, and LR: low risk.

**Table 2:** Allelic associations of SNVs in miRNA machinery genes with CML.

Gene	SNV	Alleles	Cases Freq (*n*)	Controls Freq (*n*)	*p*-Value	OR (CI 95%)
*GEMIN3*	rs197388	A	0.92 (538)	0.91 (878)		
		T	0.08 (46)	0.09 (84)	0.557	0.89 (0.614–1.301)
*DROSHA*	rs10719	G	0.67 (396)	0.65 (628)		
		A	0.33 (196)	0.35 (342)	0.385	0.91 (0.732–1.128)
	rs6877842	G	0.90 (535)	0.90 (874)		
		C	0.10 (57)	0.10 (96)	0.862	0.97 (0.687–1.370)
*XPO5*	rs11077	T	0.61 (358)	0.63 (614)		
		G	0.39 (232)	0.37 (356)	0.300	1.12 (0.905–1.382)
	rs34324334	C	0.94 (556)	0.93 (906)		
		T	0.06 (36)	0.07 (64)	0.685	0.92 (0.601–1.397)
*AGO2*	rs2293939	G	0.78 (463)	0.79 (764)		
		A	0.22 (129)	0.21 (204)	0.737	1.04 (0.813–1.338)
	rs4961280	C	0.67 (397)	0.63 (607)		
		A	0.33 (193)	0.37 (363)	0.059	0.81 (0.655–1.009)
*DICER*	rs3742330	A	0.83 (492)	0.82 (797)		
		G	0.17 (98)	0.18 (173)	0.535	0.92 (0.699–1.205)
	**rs13078**	T	0.91 (539)	0.94 (915)		
		**A**	**0.09 (53)**	**0.06 (55)**	**0.013**	**1.64 (1.105–2.421)**
*GEMIN4*	rs7813	A	0.70 (414)	0.71 (685)		
		G	0.30 (178)	0.29 (285)	0.773	1.03 (0.826–1.292)
	rs2740349	T	0.81 (477)	0.80 (776)		
		C	0.19 (115)	0.20 (194)	0.782	0.96 (0.745–1.247)
*DGCR8*	rs720012	G	0.57 (334)	0.55 (533)		
		A	0.43 (256)	0.45 (437)	0.521	0.93 (0.760–1.149)
*TNRC6B*	rs9611280	G	0.98 (578)	0.97 (937)		
		A	0.02 (14)	0.03 (33)	0.244	0.69 (0.364–1.296)

Bold letters indicate significant associations.

**Table 3:** Genotypic association of SNVs in miRNA machinery genes with CML.

Gene	SNV	Alleles	Genotype	Cases Freq (*n*)	Controls Freq (*n*)	*p*-Value	OR (CI 95%)
*GEMIN3*	rs197388	A/T	AA	0.86 (250)	0.84 (404)		
			AT	0.13 (38)	0.15 (70)	0.546	0.88 (0.569–1.339)
			TT	0.01 (4)	0.01 (7)	1.00	0.94 (0.235–3.220)
*DROSHA*	rs10719	G/A	GG	0.45 (134)	0.43 (208)		
			GA	0.43 (128)	0.44 (212)	0.680	0.94 (0.688–1.277)
			AA	0.12 (34)	0.13 (65)	0.383	0.81 (0.505–1.294)
	rs6877842	G/C	GG	0.811 (240)	0.82 (396)		
			GC	0.186 (55)	0.17 (82)	0.598	1.11 (0.756–1.612)
			CC	0.003 (1)	0.01 (7)	0.269	0.26 (0.010–1.540)
*XPO5*	rs11077	T/G	TT	0.37 (108)	0.41 (197)		
			TG	0.48 (142)	0.45 (220)	0.310	1.18 (0.859–1.616)
			GG	0.15 (45)	0.14 (68)	0.406	1.21 (0.771–1.880)
	rs34324334	C/T	CC	0.89 (263)	0.87 (424)		
			CT	0.10 (30)	0.12 (58)	0.445	0.84 (0.518–1.326)
			TT	0.01 (3)	0.01 (3)	0.680	1.61 (0.276–9.437)
*AGO2*	rs2293939	G/A	GG	0.62 (183)	0.62 (301)		
			GA	0.33 (97)	0.34 (162)	0.924	0.99 (0.720–1.344)
			AA	0.05 (16)	0.04 (21)	0.512	1.26 (0.627–2.470)
	rs4961280	C/A	CC	0.47 (138)	0.40 (193)		
			CA	0.41 (121)	0.45 (221)	0.0925	0.77 (0.561–1.046)
			AA	0.12 (36)	0.15 (71)	0.139	0.71 (0.446–1.117)
*DICER1*	rs3742330	A/G	AA	0.69 (205)	0.68 (329)		
			AG	0.28 (82)	0.29 (139)	0.741	0.95 (0.683–1.308)
			GG	0.03 (8)	0.03 (17)	0.520	0.76 (0.303–1.763)
	**rs13078**	**T/A**	TT	0.855 (253)	0.889 (431)		
			TA	0.111 (33)	0.109 (53)	0.802	1.06 (0.663–1.679)
			**AA**	**0.034 (10)**	**0.002 (1)**	**0.0004**	**15.06 (2.824–373.6)**
*GEMIN4*	rs7813	A/G	AA	0.51 (150)	0.50 (244)		
			AG	0.38 (114)	0.41 (197)	0.70	0.94 (0.691–1.280)
			GG	0.11 (32)	0.09 (44)	0.509	1.18 (0.713–1.947)
	rs2740349	T/C	TT	0.66 (196)	0.65 (313)		
			TC	0.29 (85)	0.31 (150)	0.541	0.91 (0.656–1.246)
			CC	0.05 (15)	0.04 (22)	0.806	1.09 (0.540–2.150)
*DGCR8*	rs720012	G/A	GG	0.33 (97)	0.31 (149)		
			GA	0.47 (140)	0.48 (235)	0.599	0.92 (0.658–1.275)
			AA	0.20 (58)	0.21 (101)	0.551	0.88 (0.583–1.332)
*TNRC6B*	rs9611280	G/A	GG	0.95 (282)	0.93 (452)		
			GA	0.05 (14)	0.07 (33)	0.237	0.68 (0.348–1.280)
			AA	0 (0)	0 (0)	NA	NA

Bold letters indicate significant associations.

**Table 4:** Variants in components of miRNA biosynthesis process and CML prognosis.

Gene/SNV	Allele	Sokal			Hasford			Eutos		
		HR *n* = 52	LR-IR *n* = 66	OR	HR *n* = 48	LR-IR *n* = 70	OR	HR *n* = 50	LR *n* = 47	OR
*GEMIN3* rs197388	T	0.08	0.05	1.83	0.07	0.04	1.77	0.08	0.04	2.00
*DROSHA* rs10719 rs6877842										
	A	0.28	0.36	0.7	0.27	0.34	0.71	0.28	0.36	0.68
	C	0.12	0.08	1.44	0.11	0.09	1.38	0.10	0.09	1.07
*XPO5* rs11077 rs34324334										
	G	0.44	0.36	1.39	0.47	0.36	1.54	0.42	0.42	1.01
	T	0.06	0.03	1.96	0.04	0.04	0.97	0.05	0.05	0.96
*AGO2* rs2293939 rs4961280										
	A	0.2	0.2	1.03	0.21	0.19	1.10	0.21	0.18	1.24
	A	0.37	0.33	1.15	0.33	0.37	0.85	0.34	0.40	0.79
*DICER1* rs3742330 rs13078										
	G	0.13	0.17	0.78	0.15	0.16	0.92	0.16	0.17	0.95
	A	0.08	0.11	0.7	0.09	0.09	1.01	0.09	0.09	0.96
*GEMIN4* rs7813 rs2740349										
	**G**	0.36	0.26	1.59	**0.39**	**0.24**	1.96 *	0.35	0.26	1.53
	**C**	0.25	0.17	1.67	**0.27**	**0.14**	2.23 **	0.23	0.17	1.49
*DGCR8* rs720012	A	0.6	0.56	1.16	0.59	0.55	1.20	0.58	0.56	1.07
*TNRC6B* rs9611280	A	0.02	0.02	0.84	0.03	0.01	2.23	0.02	0.02	0.96

Bold letters indicate significant associations. * *p*-value 0.019; CI 95% (1.11–3.44); ** *p*-value 0.015; CI 95% (1.16–4.28). HR: high risk, IR: intermediate risk, and LR: low risk.

**Table 5:** Linear regression estimating age at diagnosis in years.

Gene	SNV	Minor Allele	*p*-Value	β	CI 95%
*GEMIN3*	rs197388	T	0.705	−1.077	(−6.64–4.48)
*DROSHA*	rs10719	A	0.544	−0.9741	(−4.11–2.16)
	rs6877842	C	0.926	0.2497	(−5.02–5.52)
*XPO5*	rs11077	G	0.306	−1.564	(−4.55–1.42)
	rs34324334	T	0.557	−1.841	(−7.97–4.29)
*AGO2*	rs2293939	A	0.678	0.788	(−2.93–4.51)
	rs4961280	A	0.781	−0.4242	(−3.41–2.56)
*DICER1*	rs3742330	G	0.460	−1.554	(−5.67–2.56)
	rs13078	A	0.162	3.106	(−1.23–7.45)
*GEMIN4*	rs7813	G	0.059	2.955	(−0.09–6.00)
	**rs2740349**	**C**	**0.003**	**5.395**	**(1.88–8.91)**
*DGCR8*	rs720012	A	0.296	−1.589	(−4.56–1.38)
*TNRC6B*	rs9611280	A	0.301	5.233	(−4.66–15.12)

Bold letters indicate significant associations. β-value: age of diagnosis in years.

## Data Availability

The original contributions presented in this study are included in the article, further inquiries can be directed to the corresponding author.
